# Comparison between Culture Conditions Improving Growth and Differentiation of Blood and Bone Marrow Cells Committed to the Endothelial Cell Lineage

**DOI:** 10.1007/s12575-009-9023-y

**Published:** 2010-02-06

**Authors:** Claudio Muscari, Chiara Gamberini, Ilaria Basile, Francesca Bonafé, Simond Valgimigli, Ombretta Capitani, Carlo Guarnieri, Claudio Marcello Caldarera

**Affiliations:** 1 Department of Biochemistry, University of Bologna and National Institute for Cardiovascular Research, Bologna, Italy; 2 Veterinary Clinical Department, University of Bologna, Bologna, Italy; 3 Dipartimento di Biochimica "G. Moruzzi", Universita' di Bologna, Via Irnerio 48, 40126, Bologna, Italy

**Keywords:** Blood Cells, Bone Marrow Cells, Cell Proliferation, Cell Survival, Mesenchymal Stem Cells, Physiologic Neovascularization

## Abstract

The aim of this study was to compare different cell sources and culture conditions to obtain endothelial progenitor cells (EPCs) with predictable antigen pattern, proliferation potential and in vitro vasculogenesis. Pig mononuclear cells were isolated from blood (PBMCs) and bone marrow (BMMCs). Mesenchymal stem cells (MSCs) were also derived from pig bone marrow. Cells were cultured on fibronectin in the presence of a high concentration of VEGF and low IGF-1 and FGF-2 levels, or on gelatin with a lower amount of VEGF and higher IGF-1 and FGF-2 concentrations. Endothelial commitment was relieved in almost all PBMCs and BMMCs irrespective of the protocol used, whilst MSCs did not express a reliable pattern of EPC markers under these conditions. BMMCs were more prone to expand on gelatin and showed a better viability than PBMCs. Moreover, about 90% of the BMMCs pre-cultured on gelatin could adhere to a hyaluronan-based scaffold and proliferate on it up to 3 days. Pre-treatment of BMMCs on fibronectin generated well-shaped tubular structures on Matrigel, whilst BMMCs exposed to the gelatin culture condition were less prone to form vessel-like structures. MSCs formed rough tubule-like structures, irrespective of the differentiating condition used. In a relative short time, pig BMMCs could be expanded on gelatin better than PBMCs, in the presence of a low amount of VEGF. BMMCs could better specialize for capillary formation in the presence of fibronectin and an elevated concentration of VEGF, whilst pig MSCs anyway showed a limited capability to differentiate into the endothelial cell lineage.

## 1. Introduction

Endothelial progenitor cells (EPCs) promote vasculogenesis and/or ameliorate the process of angiogenesis, thereby improving both regeneration and function of ischemic organs such as the infarcted heart [1,2]. EPCs can be easily isolated from peripheral blood or bone marrow [3,4], expanded ex vivo, and then transplanted in the damaged tissues. Superficial antigens including CD34, CD133, and VEGFR-2 are often utilized to recognize EPCs during their immature stage, whilst other markers such as VE-cadherin and CD31 characterize the lineage progression towards an increased differentiation into endothelial cells [5,6].

In order to obtain EPCs, three fundamental strategies are usually followed: (1) identifying EPCs by markers which are already present in the putative immature endothelial stem cells (haemangioblast) [7], (2) selecting EPCs by their ability to form endothelial-cell like clusters [8], and (3) exploiting the plasticity of mononuclear cells (MNCs) [9,10], the latter two conditions demanding the presence of specific differentiating growth factors. The first strategy represents the only way to immediately isolate EPCs after sample collection. However, the isolation output is low and it takes a long time to reach a critical cell number useful for clinic applications. In contrast, a greater number of EPCs can be more rapidly obtained from MNCs which can generate two different subpopulations, the early and the late EPCs [11,12]. The former are usually described as MNCs which are forced to express endothelial markers within a few days; their number is elevated after isolation but these cells cannot be further expanded and they incorporate poorly into new vessels. Despite these apparent negative features, early EPCs efficiently promote in vivo neoangiogenesis especially by producing specific growth factors and chemokines. On the other hand, the number of late EPCs is extremely low after isolation and they begin to proliferate only 2–3 weeks after seeding, showing by that time an elevated proliferation potential and a satisfying ability for generating new vessels in vivo.

Although many characteristics regarding EPC isolation and culture have been already described, some aspects still need to be better defined: (1) the biological source of EPCs, since bone marrow contains a number of immature progenitors which is 500-fold higher compared to peripheral blood [13]; (2) the choice between MNCs and mesenchymal stem cells (MSCs), because the latter have been described to induce neovascularization by releasing angiogenic factors [14] and differentiating into endothelial cells [15]; and (3) the culture medium to be selected, due to the complexity of the compounds which are dissolved therein and their variable ability to stimulate differentiation and/or proliferation of cells [16].

The aim of this paper was to investigate and compare different cell sources and culture conditions in order to obtain EPCs with predictable antigen pattern and proliferation features, principally addressed to therapeutic neovascularization. With a regard to successive in vivo applications, for our study we chose the swine model which is increasingly used to investigate cardiovascular pathologies due to its anatomical analogies with humans [17].

## 2. Material and Methods

### 2.1. Cell Isolation and Treatment

Animals received adequate care in accordance with the "Principle of laboratory animal care" (NIH publication N0. 86-23, revised 1985) and with the specific Italian law (DL-116, 27 January 1992) which complies with the guide for the care and use of laboratory animals.

#### 2.1.1. Peripheral Blood-Derived Mononuclear Cells (PBMCs)

Two-month-old female pigs weighing 30–35 kg were sedated with a zolazepam–tiletamine mixture; total anaesthesia was induced with propofol and then maintained with isoflurane, fentanyl, and atracurium besylate. Pigs were placed in a supine position and the right internal jugular vein was surgically isolated. About 40 ml of blood were then collected and introduced into a bag containing a citrate phosphate dextrose adenine (CPDA) solution in a 9 to 1 blood to preserver ratio. PBMCs were isolated from venous blood by density centrifugation on Ficoll gradient (Ficoll-Paque^®^ plus; GE Healthcare, Uppsala, Sweden). PBMCs were then plated on culture dishes coated with 5 μg/ml human fibronectin or 1.5% gelatin. The PBMCs plated on fibronectin-coated dishes were maintained in Medium 119 (M119), plus penicillin and streptomycin, and supplemented with essential amino acids, 10% fetal calf serum (FCS), 50 ng/ml vascular endothelial growth factor (VEGF), 1 ng/ml human fibroblast growth factor-2 (FGF-2), and 2 ng/ml human insulin-like growth factor-1 (IGF-1). All growth factors were from Peprotech, Rocky Hill, NJ, USA. This experimental condition will be referred to hereafter as fibronectin culture condition, and the overall components and substrates as fibronectin medium.

The PBMCs which were plated on gelatin-coated dishes were maintained in endothelial cell basal medium supplemented with EGM-2 (PromoCell, Heidelberg, Germany) containing: hydrocortisone (200 ng/ml culture medium solution), 2% FCS, 0.5 ng/ml VEGF, 10 ng/ml human FGF-2, 5 ng/ml human epidermal growth factor (EGF), 20 ng/ml human IGF-1, and 1 μg/ml ascorbic acid. The supplemented endothelial cell growth medium was integrated with essential amino acids plus penicillin and streptomycin. This enriched EGM-2 solution should promote cell proliferation better than cell differentiation with respect to the fibronectin medium, due to the higher concentrations of FGF-2 and IGF-1 together with EGF. This experimental condition will be referred to hereafter as gelatin culture condition, and the overall components and substrates as gelatin medium.

#### 2.1.2. Bone Marrow-Derived Mononuclear Cells (BMMCs)

Pigs were placed on their right side and 50 ml of bone marrow were aspirated from the ileal crest and mixed at a 1:1 volume ratio with M199 containing 5% sodium heparin. The sample was filtered onto a 100-μm nylon cell strainer (BD Falcon, Franklin Lakes, NJ, USA) and the mononuclear cell fraction (BMMCs) was isolated by density centrifugation on Ficoll gradient. BMMCs were exposed either to the fibronectin or gelatin culture condition. Some experiments were carried out by growing BMMCs under an intermediate culture condition consisting of fibronectin-coated dishes with the EGM-2 supplemented solution [18,19].

#### 2.1.3. Mesenchymal Stem Cells (MSCs)

BMMCs were enriched in MSCs by growing them on plastic for about 2 weeks. Pig bone marrow was aspirated according to the above-mentioned procedure described for BMMC isolation. Cells were filtered through a 100-μm nylon filter (BD Falcon) and plated into a 75-cm^2^ flask. Cells were then grown in complete αMEM containing 10% fetal bovine serum (FBS), 2 mM L-glutamine, 100 U/ml penicillin, and 100 μg/ml streptomycin at 37°C and 5% CO_2_ for 3 days. The medium was then replaced with fresh medium and the adherent cells were grown for further 10–12 days (MSCs), reaching about 90% confluence.

MSCs were cultured under both fibronectin and gelatin culture conditions for 1 week and then investigated for changes in cell antigens and angiogenic functions. More detailed morphologic and immunophenotypic properties of bone-derived MSCs have been described in our previous work [20,21].

### 2.2. Cytochemical Characterization

Direct fluorescent staining was used to detect the binding of fluorescein isothiocyanate (FITC)-conjugated BS-I, a lectin obtained from Bandeiraea simplicifolia (Sigma, St. Louis, Missouri, USA) [22] and cell uptake of 1,1-dioctadecyl-3,3,3,3-tetramethilindocarbocyanine (Dil)-labelled acetylated low density lipoprotein (acLDL; Molecular Probes, Eugene, Oregon, USA). Cells were incubated with 10 μg/ml acLDL for 4 h and with 10 μg/ml BS-I for 2 h at 37°C and finally fixed with 3% paraformaldehyde in PBS for 15 min.

Regarding the immunofluorescence analysis, cells were grown on glass coverslips under the differentiating conditions, fixed with 3% paraformaldehyde, and rinsed twice with PBS. After blocking with 4% BSA and 0.2% Tween 20 in PBS for 1 h at room temperature, the cells were incubated with primary mouse monoclonal antibodies against the following antigens: human VEGFR-2 1:20 (MAB3571; R&D Systems, Minneapolis, MN, USA; its cross-reactivity with pig VEGFR-2 was verified in the present study); pig CD31 1:10 (MCA1746; AbD Serotec, Düsseldorf, Germany); pig macrophages 1:100 (MCA2317; AbD Serotec); human CD90 1:100 (550402; BD Bioscience, Pharmingen, San Diego, CA, USA; cross-reactive with pig CD90). All primary antibodies were diluted in the blocking reagent. FITC-conjugated anti-mouse antibodies (Sigma) were used as secondary antibodies (diluted in blocking buffer 1:2000). After immunofluorescence staining, cells were mounted with the bleaching reagent (ProLong Gold antifade reagent; Molecular Probes) on standard glass slide and observed by an inverted fluorescence microscope (Olympus IX50).

Human umbilical vein endothelial cells (HUVECs; Lonza, Warkerville, MD, USA) were chosen as positive control cells either for direct fluorescence staining (Dil-acLDL, FITC-UEA-1) or immunofluorescence analysis. More than 85% of HUVECs was positively stained by the above-mentioned endothelial markers (data not shown). H9c2 rat cardiomyoblasts (ECACC) were used as negative control cells. They were substantially not stained by the endothelial markers, since at most 2.5% of them showed target-specific fluorescence (data not shown).

The percentage of cells staining positive to each fluorescent markers was calculated using ten randomly selected high-power fields for three separate experiments.

### 2.3. Cell Seeding and Cell Adhesion on a Hyaluronan-Based Scaffold

The BMMCs were grown until confluence on coated dishes under fibronectin-, gelatin-, or intermediate culture conditions, then detached by incubation with 1% EDTA solution in PBS for 10 min at 37°C and counted in duplicate using a Burker hemocytometer. Some aliquots of post-confluent BMMCs were re-seeded on the coated dish under the same condition for 1 week to obtain first passage cells. BMMCs were suspended in M199 and slowly dispensed at the density of 1 × 10^4^ cells/mm^3^ onto three-dimensional pieces of HYAFF^®^11 (Fidia Advanced Biopolymers, Abano Terme, Italy), a non-woven hyaluronan-based scaffold (surface: 2–10 mm^2^; height: 1 mm), which were lodged in a 24-well cell culture plate [23]. Cells were allowed to attach to the scaffold for 3 h at 37°C before adding the complete culture medium (M199 with growth factors or EGM-2).

The trypan blue exclusion staining method was used to quantify cell adhesion into the biopolymer after 24 h from seeding. The number of cells which adhered to the fibers was calculated by subtracting the number of cells attached on the well bottom and those still in suspension from the total amount of seeded cells:

$$\begin{array}{c} \text{HYAFF-adherent cells}=\text{total seeded cells}\\ -\text{well attached and suspended cells} \end{array}$$

### 2.4. Cell Proliferation and Viability

Cell expansion was measured by the trypan blue exclusion staining method which allows the quantification of living cells by counting them in a Bürker's counting chamber. Cell viability was assessed by using the Alamar Blue dye (BioSource, Camarillo, CA), a nontoxic aqueous compound formulated to quantify viability and proliferation of living cells. This method consists in the indicator reduction into a fluorescent form in response to changes in the mitochondrial redox activity. In brief, adherent cells or cells seeded on the scaffold were incubated after medium removal at 37°C for 4 h with 1 ml of Alamar Blue solution (10% v/v in complete culture medium) according to the manufacter's instructions. Alamar Blue reduction was quantified in triplicate from each sample (Ex 540 nm/Em 590 nm) using a Wallac VICTOR^2^ multiwell reader (Perkin Elmer, Milan, Italy).

### 2.5. In Vitro Vasculogenesis

The assay was performed on Matrigel (BD Biosciences, Bedford, MA, USA) according to manufacturer's instructions. Briefly, ECmatrix™ solution was placed in a 96-well tissue culture plate at 37°C for 1 h to allow the matrix solution to solidify. BMMCs or MSCs were treated with the above-mentioned endothelial differentiating media and successively detached with 1 mM EDTA/PBS at 37°C for 20 min, harvested by centrifugation, and re-plated alone (2.0 × 10^4^ cells/well) or together with HUVECs (2.0 × 10^4^ cells/well) on the solidified matrix solution. Cells seeded on Matrigel were incubated in M199 at 37°C for 24 h and tubule-like formation was then inspected under an inverted light microscope (Olympus IX50).

### 2.6. Statistical Analysis

Values are expressed as the mean ± standard error of the mean. The statistical analysis was performed by unpaired Student's *t* test. *P* < 0.05 was considered significant.

## 3. Results

### 3.1. Experiments with PBMMCs and BMMCs

#### 3.1.1. PBMMC and BMMC Commitment to the Endothelial Cell Lineage

Almost all PBMCs were positive to both acLDL uptake and BS-I binding after just 1 week of fibronectin culture condition (Table 1). These two markers were readily detectable in more than 90% of cells even only 3 weeks after cell seeding. VEGFR-2 was expressed by about 75% of PBMCs after 1 week and by 95% of cells after 2 and 3 weeks. In contrast, the mature endothelial cell marker CD31 and the macrophage antigen were present only at a low percentage in PBMCs throughout the experiment. Moreover, CD90 was not expressed suggesting that adherent PBMCs were not oriented toward the mesenchymal lineage.

**Table 1:** Antigen pattern of PBMCs and BMMCs cultured under endothelial differentiating conditions

	PBMC/F			PBMC/G			BMMC/F = BMMC/G		
			
	d7	d14	d21	d7	d14	d21	d7	d14	d21
AcLDL	4+	4+	4+	4+	3+	2+	4+	3+	1+
BS-I	4+	4+	4+	4+	3+	3+	4+	4+	4+
VEGFR-2	3+	4+	4+	4+	3+	3+	4+	4+	4+
CD31	1+	1+	1+	1+	1+	1+	1+	1+	1+
Macrophage	1+	1+	1+	1+	1+	1+	1+	1+	1+
CD90	-	-	-	-	-	-	-	-	-

Cells were observed by use of an inverted fluorescence microscope after 7, 14, and 21 days under fibronectin (*F*) or gelatin (*G*) culture condition. Cell counting was performed by calculating the cell average number of 10 randomly selected high-power fields (×200) of 3 separate experiments

AcLDL and BS-I were Dil and FITC conjugated, respectively. Immunofluorescence of VEGFR-2, CD31, macrophage antigen, and CD90 was detected by FITC-conjugated polyclonal secondary antibodies. The pattern of markers evaluated for BMMCs gave the same results irrespective of the culture condition

Fluorescence scores:* 4+* >90%,* 3+* 50–90%*, 2+* 20–50%,* 1+* <20%, - no fluorescence

Nearly all PBMCs exposed to the gelatin medium for 1 week were positive stained by the endothelial markers, with the exception of CD31, although a general reduction in the expression of the endothelial antigens and the acLDL uptake was observed after 2 weeks (Table 1).

More than 95% of BMMCs committed to the pre-endothelial cell phenotype under the fibronectin culture condition after just 1 week and maintained the pattern of endothelial markers up to the third week (Table 1). A similar behavior was observed for BMMCs exposed to the gelatin medium. Only the uptake of acLDL decreased after the second week, independently of the medium used; this was probably related to the detachment and re-plating of confluent BMMCs that can be responsible for partial damage of the scavenger receptor.

#### 3.1.2. PBMMC and BMMC Proliferation and Viability under Endothelial Cell Differentiating Conditions

The ability of PBMC to expand was very low, irrespective of the culture medium. In particular, PBMCs cultured on fibronectin-coated dishes never did reach confluence throughout the study. Cell confluence was observed only in 30% of dishes under the gelatin culture condition and, in any case, not before 2 weeks from cell seeding (Table 2). Post-confluent PBMCs did not keep proliferating.

**Table 2:** Comparison between the proliferation potential of treated PBMCs and BMMCs

	PBMC/F	PBMC/G	BMMC/F	BMMC/G
% dishes with confluent cells	0	30	60	100
Confluence time		>2 wk	<2 wk	<2 wk
% increase in 1st passage cell-number		0	50	70
% increase in 2nd passage cell-number			20	50

The trypan blue exclusion staining was performed to quantify cells which were grown under fibronectin (*F*) or gelatin (*G*) culture condition. Re-seeding was carried out when cells reached confluence. Cell counting was performed by calculating the cell average number of 10 randomly selected high-power fields (×200) of 5 separate experiments. The percentage increase in the post-confluent cell number was calculated 1 week after re-seeding

Differently from PBMCs, BMMCs showed a high proliferation rate, especially with the gelatin medium (Table 2). BMMCs mostly reached confluence in a shorter time with respect to PBMCs. Moreover, BMMCs became rapidly confluent even after the second passage.

**Figure 1:**
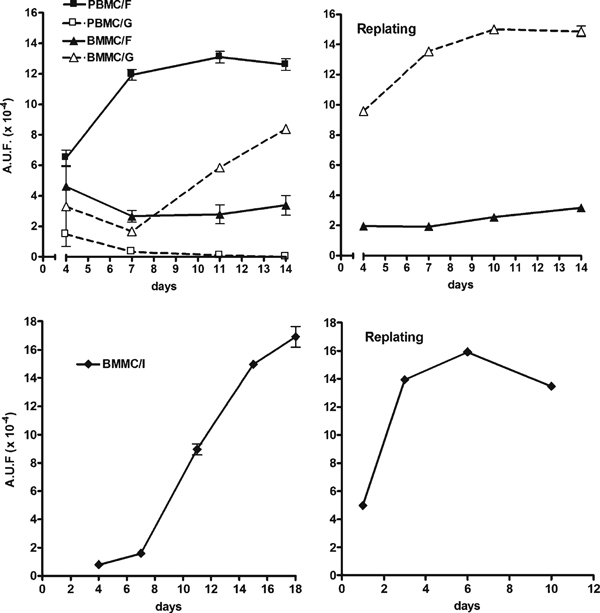
**Time-course of PBMC and BMMC viability exposed to endothelial differentiating conditions**. Cell viability was assessed by the Alamar Blue assay as described in the Methods section. Plots are representative of 5 separate experiments performed in triplicate. *A.U.F.* Arbitrary units of fluorescence. *Upper diagrams.* Plots on the *left* show the time-course of cells seeded just after isolation and never detached from the culture dish till confluence. Plots on the *right* show the behavior of cell re-seeding following the first confluence. Cells were grown under fibronectin (*F*) or gelatin (*G*) culture conditions. *Lower diagrams.* The viability of BMMCs grown under the intermediate culture condition (*I*) is shown before (*left*) and after (*right*) first-passage re-seeding.

In contrast, PBMCs grown in the fibronectin medium were more viable than those cultured in the gelatin medium, as evaluated by the Alamar blue test (Figure 1, left upper diagrams).

BMMCs treated with the fibronectin medium maintained their viability constant throughout the experiment, even after cell replating (Figure 1, left upper diagrams), whereas BMMCs cultured on gelatin-coated dishes increased their viability over time, even after the first passage. The positive effect on BMMC viability observed under the gelatin culture condition was probably enhanced by the presence of an elevated number of cells which, thanks to their own paracrine mitogenic function [24], exhibited a high rate of proliferation (Table 2).

In order to understand which component of the gelatin culture condition was responsible for cell viability, we differently combined some characteristics of the two formulations, by growing BMMCs on fibronectin-coated dishes supplemented with the EGM-2 solution (Figure 1, lower diagrams). Under this intermediate culture condition, the viability of BMMCs rapidly increased within the first 18 days and showed a further increment up to the first week after cell re-seeding. Therefore, we suggest that the improved viability of BMMCs was dependent on the specific cocktail of growth factors which were present in the gelatin medium rather than gelatin in itself.

Both proliferation potential and viability were also investigated in those BMMCs which were suspended in the cell medium during the first 3 days of culture. This study was performed because these cells are sometimes evaluated for their ability to generate endothelial cells. The behavior of these cells was similar to that of the early-adherent BMMCs, although their rate of proliferation and their viability were inferior (*n* = 4, data not shown).

#### 3.1.3. Adhesion and Viability of BMMC-Derived EPCs Cultured on a Hyaluronan-Based Scaffold

**Figure 2:**
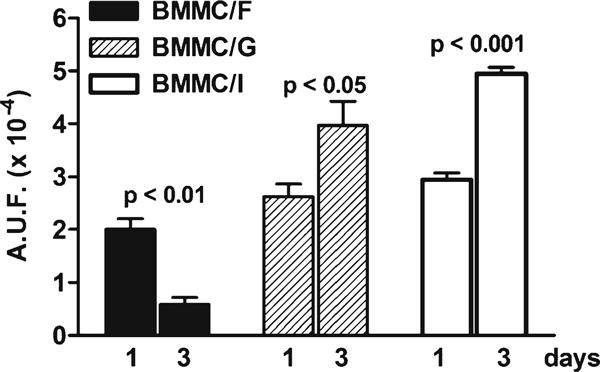
**Viability of treated BMMCs cultured on HYAFF^®^11**. The viability of endothelial-committed BMMCs was evaluated by the Alamar Blue assay during the first 3 days of cell growth on HYAFF^®^11. Cell pre-tretment was performed under fibronectin (*F*), gelatin (*G*), or intermediate (*I*) culture condition.* Bar graphs* are representative of 3 separated experiments performed in triplicate.* A.U.F.* Arbitrary units of fluorescence.

Figure 2 shows the early changes in viability of BMMCs under fibronectin-, gelatin-, and intermediate culture conditions grown on HYAFF^®^11. We previously described the ultrastructural features of this scaffold and the properties of human eEPCs cultured onto its fibers [23]. The percentage of pig BMMCs adhesion to the fibers after 24 h from seeding was more than 95% under all culture conditions. Cell adhesion was evaluated in post-confluence BMMCs and was higher than 90% (data not shown). A few cells which did not adhere to the fibers were positive to trypan blue staining and remained suspended in the culture medium, whilst others remained layered on the bottom of the well.

The viability of BMMCs pre-cultured in the fibronectin medium markedly decreased after 3 days from seeding on the scaffold. In contrast, BMMCs which were pre-treated with gelatin or intermediate medium significantly increased their viability. This confirms that EPCs derived from BMMCs show a better metabolism when cultured with the EGM-2 solution even when seeded on a polymer scaffold.

#### 3.1.4. BMMC-Induced Formation of Tubule-Like Structures

**Figure 3:**
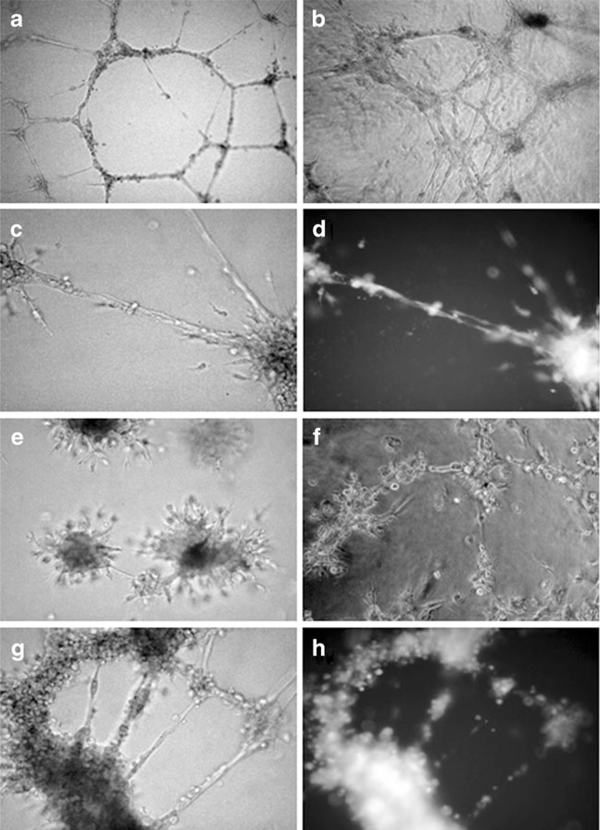
**In vitro tubule-like structure formation driven by pre-treated BMMCs**. The micrographs are representative of 3 separate experiments. HUVECs alone generated well-defined capillary-like structures on Matrigel (**a**, ×100. BMMCs pre-treated with the fibronectin medium and seeded on Matrigel formed a similar network either alone (**b**, ×100) or with HUVECs (**c,d**). These co-cultures were observed under phase contrast (**c**) or fluorescence (**d**) microscopy to evidence CFDA-labelled BMMCs (×200). When BMMCs were pre-treated with the gelatin medium, they produced single agglomerates (**e**, ×100) or interrupted rings (**f**, ×100). BMMCs treated with the gelatin medium developed networks when cultured with HUVECs (**g**, phase contrast; **h**, fluorescence of FITC-BS-I-labeled BMMCs).

We performed the standard test on Matrigel to investigate cell ability to generate vessel-like structures. HUVECs were used either as a positive control (alone) or as a cellular network to sustain the angiogenic activity of the EPCs used in the present work. BMMCs pre-treated with the fibronectin medium generated well-defined tubule-like structures (Figure 3b) which were similar to those formed by HUVECs alone (Figure 3a). In addition, the ability of BMMCs to integrate with the HUVEC network was satisfactory, since cell rings were well surrounded and intercalated by FITC-BS-I-labelled BMMCs (Figure 3c,d).

BMMCs exposed to the gelatin culture condition were less prone to form vessel-like structures. Some of them remained isolated as single cell aggregates, spreading limited cell extensions along radial directions (Figure 3e). Moreover, they did not always originate completely interconnected rings (Figure 3f) although FITC-BS-I-labeled BMMCs could intercalate themselves in the HUVEC network (Figure 3g, h).

### 3.2. Experiments with MSCs

#### 3.2.1. MSC Commitment to the Endothelial Cell Lineage

We also investigated the commitment to endothelial cells of MSCs, a naturally occurring stem cell population which resides in bone marrow and other tissues [25]. Table 3 shows that untreated MSCs (day 0) were markedly positive for CD90; this finding is in agreement with a previous report [26]. In contrast, these cells were completely negative for the macrophage antigen. The uptake of acLDL was also not reported in the untreated MSCs, while BS-I binding and VEGFR-2 antigen expression were observed only in 10% MSCs. After 1-week exposure to the fibronectin medium, 10–20% of MSCs were otherwise able to uptake acLDL and bind BS-I, while about 50% of MSCs expressed VEGFR-2. A parallel and complete disappearance of CD90 was also observed after 1 week. Similar changes were induced in MSCs under the gelatin culture condition.

**Table 3:** Phenotypic changes of MSCs exposed to endothelial differentiating conditions

	Day 0	Day 7 (F = G)
AcLDL	-	1+
BS-I	1+	1+
VEGFR-2	1+	2+
CD31	1+	1+
Macrophage	-	-
CD90	3+	-

MSCs were labelled with the fluorescent dyes described in the legend of Table 1 and observed under fluorescence microscopy. MSCs were grown under standard basal condition (*Day 0*) and under fibronectin (*F*) or gelatin (*G*) culture condition for 7 days (*Day 7*). There were no substantial differences between MSCs cultured with these two different media (*F = G*)

Cell counting was performed by calculating the cell average number of 10 randomly selected high-power fields (×200) of 3 separated experiments

Fluorescence score:* 3+* 50–90%,* 2+* 20–50%,* 1+* <20%, - no fluorescence

#### 3.2.2. MSC-Induced Formation of Tubule-Like Structures

**Figure 4:**
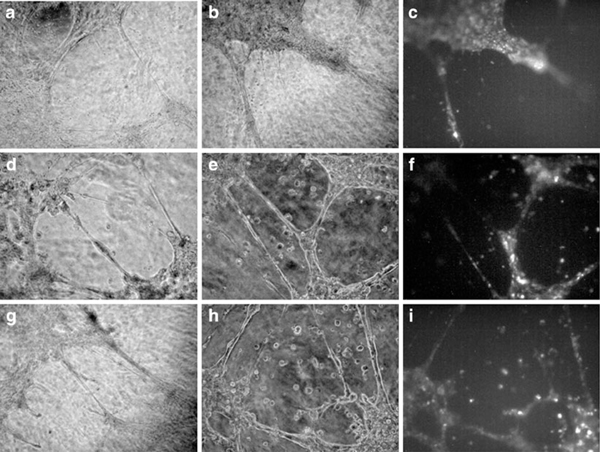
**In vitro tubule-like structure formation by untreated MSCs or MSCs cultured with endothelial differentiating media**. Untreated MSCs were cultured alone (**a**) or with HUVECs (**b,c**). MSCs pre-treated with fibronectin or gelatin medium were also cultured alone (**d** and **g**, respectively) or with HUVECs (**e**,**f** and **h**,**i**, respectively). FITC-UEA-1-labeled HUVECs co-cultured with MSCs are shown in (**c**), (**f**) and (**i**). The micrographs are representative of triplicate experiments performed using MSCs on Matrigel.

Despite the negligible presence of endothelial markers, the untreated MSCs seeded on Matrigel were able to arrange some wide-sized rings (Figure 4a) and to partially contribute to the HUVEC-driven network (Figure 4b, c). In this case, we labeled HUVEC with the fluorescent lectin rather than MSCs since these latter did not bind it well. The ability to produce tubule-like structures was improved by using treated MSCs; in fact, irrespective of the culture condition used they originated closed rings (Figure 4d, g) and better participated in the HUVEC-mediated capillary formation (Figure 4e, f, h, i).

## 4. Discussion

Judging from the positive staining for the endothelial markers, the combination of high concentration of VEGF together with fibronectin substrate produced a reliable EPC phenotype in PBMCs which was maintained longer than under the gelatin culture condition. However, the low expression of CD31 indicates that complete maturation could not be achieved during the 3-week exposure to both differentiating media. Differently from PBMCs, more than 90% of BMMCs could bind BS-I and express VEGFR-2 up to 3 weeks under the gelatin culture condition. Thus, BMMCs are more prone than PBMCs to maintain over time an EPC phenotype when exposed to the gelatin-based differentiating conditions.

Cell proliferation was markedly affected by both the biological sources of mononuclear cells and the culture conditions. PBMCs showed a very low attitude to proliferate, although they reacted better to the gelatin medium. Only under this latter condition was it possible to obtain PBMC confluence in 30% of culture dishes, but only after 20 days since cell seeding. However, confluent cells were not able to proliferate any more. In contrast, more than 60% of BMMCs grown in the fibronectin medium could rapidly expand and reach confluence even at the second passage within 3 weeks. This finding is in agreement with the report of Mertsching et al. [27] who expanded pig BMMCs under the same culture conditions. We obtained the best proliferation performance with BMMCs exposed to the gelatin-based formulation, since in 100% dishes it was possible to re-split cells and increase cell number over 50% after 1 week from each re-seeding. Although proliferation was better induced by gelatin rather than fibronectin culture condition, the latter composition allowed PBMCs to be more viable up to the first confluence. A possible explanation may be the efficacy by which cells adhere to the culture dish, since a high number of cell contacts stimulate survival pathways in most cells (anoikis) [28]. Fibronectin is known, indeed, to bind cells through a wide pattern of integrins and other receptor-like structures, hence affecting endothelial cell viability and functions [29-31]. Moreover, VEGF was present in the fibronectin medium at a high level, thus improving cell survival [32].

In contrast, BMMC viability was positively affected by the gelatin medium based on their more elevated rate of cell proliferation. This was especially evident during the second passage when the exponential growth of BMMCs was more pronounced. To better understand which was the relative involvement of the culture components on the behaviour of BMMCs, we studied a third condition in which cells were grown on fibronectin-coated dishes in the presence of EGM-2 solution. The viability of BMMCs was similar to those obtained with the gelatin-based formulation, suggesting that these two processes were stimulated by the higher amount of IGF-1 and FGF-2, together with EGF, since they synergistically increase cell growth and survival [33,34]. The unattached BMMCs which remained suspended in the medium during the first 3 days of culture were also able to subsequently adhere to the coated dishes and expand; thus, we suggest that the amount of BMMC-derived EPCs can be further increased by simultaneously growing both early-suspended and adherent cells under the same culture conditions.

As a consequence of these preliminary results, we decided not to perform further investigations on PBMCs-derived EPCs because they showed a poor proliferative potential. We did not try to mobilize EPCs from bone marrow, i.e. by means of the granulocyte colony-stimulating factor (G-CSF), because the yield of these cells in peripheral blood is, in any case, lower than that registered by using bone marrow itself [13,35].

Among the natural polymers which are commonly used as suitable scaffolds for regenerative medicine, the hyaluronan-based materials show interesting features because they are biocompatible, biodegradable, and can stimulate biological activities through hyaluronan-related cell receptors [36]. Several approaches of cell therapy need rapid vascularization during the regeneration of the damaged tissue; in this case, hyaluronan plays a beneficial role not only as a cell vehicle but also because it can activate neoangiogenesis through the slow release of its naturally degraded fragments [37].

The percentage of the BMMC-derived EPCs which adhered to the scaffold was higher than 90%, irrespective of the differentiating medium, and even using second-passage cells. This latter finding suggests that we can expand post-confluent cells and also obtain an almost complete adhesion of these EPCs to the scaffold. In a previous report, we suggested the transplanting in vivo of HYAFF^®^11 engineered with human EPCs after just a few days from cell seeding in order to obtain the best effects on neovascularization [23]; therefore, we investigated the viability of treated BMMCs within 3 days of culture on the scaffold. As for cultures on coated dishes, the best viability was observed by pre-treating BMMCs under gelatin or intermediate culture condition. Also under these conditions, BMMC growth on a hyaluronan-base polymer was attributable to the positive effects elicited by IGF-1, FGF-2, and EGF.

The ability of BMMCs to generate a tubule-like network was evaluated using Matrigel as a standard substrate. The pre-treatment of BMMCs with the fibronectin medium allowed the formation of three-dimensional rings which were without gaps, like those derived from HUVECs. Under this condition, BMMCs were also able to plug into the HUVEC network, extending thin connections formed by single cell layers such as during the in vivo capillary formation. Therefore, BMMCs treated with the fibronectin medium showed both angiogenic- and vasculogenic-like properties in vitro. In contrast, the gelatin medium was not as efficient as the fibronectin one to prime BMMCs alone for tubule-like formation.

Taken together, these results suggest that a leading protocol to obtain a suitable number of specialized EPCs for neovascularization could be to expand BMMCs with the gelatin-based formulation and to expose them to the fibronectin medium to optimize their ability to produce a reliable capillary network. An alternative strategy was the one adopted by Serrano et al. [38], which was characterized by exposing pig PMBCs to EGM-2 for 2 weeks and then depriving the medium of FGF-2 with the purpose of enhancing endothelial differentiation in this second phase of culture.

The study of both function and differentiation of MSCs gave further useful information. First of all, MSCs alone and in the absence of any pre-treatment with endothelial differentiating media could form initial and rough tubule-like structures in Matrigel. As a matter of fact, the test we used to evaluate tubule-like formation on Matrigel is not exclusive for capillary structures, but allows the circular joining of other kinds of cells, such as fibroblasts [39]. Since native MSCs did not show a pattern of endothelial markers, it is likely that their attempt to create a cell network was mainly referred to their mesenchymal phenotype. MSCs, together with EPCs, indeed represent an excellent mixed cell population that has been described as originating capillaries with pericytes [14] and vessels of middle/large size provided with a complete vascular wall [40].

One main limitation of the present work is the lack of investigation about pig EPCs obtained by selecting an antigen expressed by very early immature precursor cells, i.e. CD34 and CD133, or by the expansion of endothelial cell-forming colony units. However, both these protocols yield few EPCs in a short time, thereby reducing their availability for precocious intervention of cardiac regeneration. Nevertheless, these cells can also be greatly expanded, but the time available to obtain a suitable number of functional EPCs for clinical applications is about 2 months. Another limit of this research is the absence of studies related to EPC migration and EPC release of angiogenic factors. The assay of in vitro tubule-like structure formation that was performed in the present work should also include these EPC activities, although caution is needed to directly correlate this morphological assay with endothelial functionality in vivo [41].

In conclusion, pig BMMCs are preferentially expanded rather than differentiated to endothelial cells using high concentrations of IGF-1 and FGF-2, in the presence of EGF and a low level of VEGF. In contrast, fibronectin-coated dishes supplemented with an elevated amount of VEGF and lower concentrations of IGF-1 and FGF-2 can be considered a good formulation to produce capillary-like structures by BMMCs. Therefore, these two different protocols could be followed and integrated to prepare a large number of specialized EPCs as a useful strategy to modulate neovascularization.

## References

[B1] Marsboom G Janssens S Endothelial progenitor cells: new perspectives and applications in cardiovascular therapies Expert Rev Cardiovasc Ther 2008 6 687 701 10.1586/14779072.6.5.687 18510485 18510485

[B2] Yamahara K Itoh H Potential use of endothelial progenitor cells for regeneration of the vasculature Ther Adv Cardiovasc Dis 2009 3 17 27 10.1177/1753944708097728 19144668 19144668

[B3] Boyer M Townsend LE Vogel LM Falk J Reitz-Vick D Trevor KT Isolation of endothelial cells and their progenitor cells from human peripheral blood J Vasc Surg 2000 31 1 Pt 1 181 189 10.1016/S0741-5214(00)70080-2 10642721 10642721

[B4] Murayama T Asahara T Bone marrow-derived endothelial progenitor cells for vascular regeneration Curr Opin Mol Ther 2002 4 395 402 12222878 12222878

[B5] Hristov M Erl W Weber PC Endothelial progenitor cells. Isolation and characterization Trends Cardiovasc Med 2003 13 201 206 10.1016/S1050-1738(03)00077-X 12837583 12837583

[B6] Urbich C Dimmeler S Endothelial progenitor cells: characterization and role in vascular biology Circ Res 2004 95 343 353 10.1161/01.RES.0000137877.89448.78 15321944 15321944

[B7] Park C Ma YD Choi K Evidence for the hemangioblast Exp Hematol 2005 33 965 970 10.1016/j.exphem.2005.06.003 16140143 16140143

[B8] Hirschi KK Ingram DA Yoder MC Assessing identity, phenotype, and fate of endothelial progenitor cells Arterioscler Thromb Vasc Biol 2008 28 1584 1595 10.1161/ATVBAHA.107.155960 18669889 18669889 PMC5244813

[B9] Fernandez Pujol B Lucibello FC Gehling UM Lindemann K Weidner N Zuzarte ML Endothelial-like cells derived from human CD14 positive monocytes Differentiation 2000 65 287 300 10.1046/j.1432-0436.2000.6550287.x 10929208 10929208

[B10] Rehman J Li J Orschell CM March KL Peripheral blood "endothelial progenitor cells" are derived from monocyte/macrophages and secrete angiogenic growth factors Circulation 2003 107 1164 1169 10.1161/01.CIR.0000058702.69484.A0 12615796 12615796

[B11] Hur J Yoon CH Kim HS Choi JH Kang HJ Hwang KK Characterization of two types of endothelial progenitor cells and their different contributions to neovasculogenesis Arterioscler Thromb Vasc Biol 2004 24 288 293 10.1161/01.ATV.0000114236.77009.06 14699017 14699017

[B12] Yoon CH Hur J Park KW Kim JH Lee CS Oh IY Kim Synergistic neovascularization by mixed transplantation of early endothelial progenitor cells and late outgrowth endothelial cells Circulation 2005 112 1618 1627 10.1161/CIRCULATIONAHA.104.503433 16145003 16145003

[B13] Kamihata H Matsubara H Nishiue T Fujiyama S Amano K Iba O Improvement of collateral perfusion and regional function by implantation of peripheral blood mononuclear cells into ischemic hibernating myocardium Arterioscler Thromb Vasc Biol 2002 22 1804 1810 10.1161/01.ATV.0000039168.95670.B9 12426208 12426208

[B14] Caplan AI Why are MSCs therapeutic? New data: new insight J Pathol 2009 217 318 324 10.1002/path.2469 19023885 19023885 PMC8793150

[B15] Oswald J Boxberger S Jørgensen B Feldmann S Ehninger G Bornhäuser M Mesenchymal stem cells can be differentiated into endothelial cells in vitro Stem Cells 2004 22 377 384 10.1634/stemcells.22-3-377 15153614 15153614

[B16] Sieveking DP Ng MK Cell therapies for therapeutic angiogenesis: back to the bench Vasc Med 2009 14 153 166 10.1177/1358863X08098698 19366823 19366823

[B17] Douglas WR Of pigs and men and research: a review of applications and analogies of the pig, sus scrofa, in human medical research Space Life Sci 1972 3 226 234 10.1007/BF00928167 4556756 4556756

[B18] Allen J Khan S Serrano MC Ameer G Characterization of porcine circulating progenitor cells: toward a functional endothelium Tissue Eng 2008 14 183 194 10.1089/ten.2007.0265 18333816

[B19] Mieno S Clements RT Boodhwani M Sodha NR Ramlawi B Bianchi C Characteristics and function of cryopreserved bone marrow-derived endothelial progenitor cells Ann Thorac Surg 2008 85 1361 1366 10.1016/j.athoracsur.2007.12.006 18355528 18355528

[B20] Muscari C Bonafe' F Farruggia G Stanic I Gamberini C Carboni M Long-term treatment with N-acetylcysteine, but not caloric restriction, protects mesenchymal stem cells of aged rats against tumor necrosis factor-induced death Exp Gerontol 2006 41 800 804 10.1016/j.exger.2006.05.003 16806781 16806781

[B21] Pasquinelli G Orrico C Foroni L Bonafè F Carboni M Guarnieri C Mesenchymal stem cell interaction with a non-woven hyaluronan-based scaffold suitable for tissue repair J Anat 2009 213 520 530 10.1111/j.1469-7580.2008.00974.x PMC2667546 19014359

[B22] Ruel M Wu GF Khan TA Voisine P Bianchi C Li J Inhibition of the cardiac angiogenic response to surgical FGF-2 therapy in a Swine endothelial dysfunction model Circulation 2003 108 Suppl 1 II335 II340 12970256 12970256 10.1161/01.cir.0000087903.75204.ad

[B23] Pasquinelli G Vinci MC Gamberini C Orrico C Foroni L Guarnieri C Architectural organization and functional features of early endothelial progenitor cells cultured in a hyaluronan-based polymer scaffold Tissue Eng Part A 2009 15 2751 2762 10.1089/ten.tea.2008.0232 19438299 19438299

[B24] He T Peterson ET Katusic ZS Paracrine mitogenic effect of human endothelial progenitor cells: role of interleukin-8 Am J Physiol Heart Circ Physiol 2005 289 H968 H972 10.1152/ajpheart.01166.2004 15805227 15805227

[B25] Rho GJ Kumar BM Balasubramanian SS Porcine mesenchymal stem cells–current technological status and future perspective Front Biosci 2009 14 3942 3961 10.2741/3503 19273325 19273325

[B26] Wang X Hu Q Mansoor A Lee J Wang Z Lee T Bioenergetic and functional consequences of stem cell-based VEGF delivery in pressure-overloaded swine hearts Am J Physiol Heart Circ Physiol 2006 290 H1393 H1405 10.1152/ajpheart.00871.2005 16387794 16387794

[B27] Mertsching H Walles T Hofmann M Schanz J Knapp WH Engineering of a vascularized scaffold for artificial tissue and organ generation Biomaterials 2005 26 6610 6617 10.1016/j.biomaterials.2005.04.048 15979139 15979139

[B28] Michel JB Anoikis in the cardiovascular system: known and unknown extracellular mediators Arterioscler Thromb Vasc Biol 2003 23 2146 2154 10.1161/01.ATV.0000099882.52647.E4 14551156 14551156

[B29] Almeida EA Ilić D Han Q Hauck CR Jin F Kawakatsu H Matrix survival signaling: from fibronectin via focal adhesion kinase to c-Jun NH(2)-terminal kinase J Cell Biol 2000 149 741 754 10.1083/jcb.149.3.741 10791986 10791986 PMC2174844

[B30] Maurice S Srouji S Livne E Isolation of progenitor cells from cord blood using adhesion matrices Cytotechnology 2007 54 121 133 10.1007/s10616-007-9077-0 19003027 19003027 PMC2267502

[B31] Prasad Chennazhy K Krishnan LK Effect of passage number and matrix characteristics on differentiation of endothelial cells cultured for tissue engineering Biomaterials 2005 26 5658 5667 10.1016/j.biomaterials.2005.02.024 15878371 15878371

[B32] Fujio Y Walsh K Akt mediates cytoprotection of endothelial cells by vascular endothelial growth factor in an anchorage-dependent manner J Biol Chem 1999 274 16349 16354 10.1074/jbc.274.23.16349 10347193 10347193 PMC3624707

[B33] Chen CH Poucher SM Lu J Henry PD Fibroblast growth factor 2: from laboratory evidence to clinical application Curr Vasc Pharmacol 2004 2 33 43 10.2174/1570161043476500 15320831 15320831

[B34] Kurmasheva RT Houghton PJ IGF-I mediated survival pathways in normal and malignant cells Biochim Biophys Acta 2006 1766 1 22 16844299 16844299 10.1016/j.bbcan.2006.05.003

[B35] Honold J Lehmann R Heeschen C Walter DH Assmus B Sasaki K Effects of granulocyte colony simulating factor on functional activities of endothelial progenitor cells in patients with chronic ischemic heart disease Arterioscler Thromb Vasc Biol 2006 26 2238 2243 10.1161/01.ATV.0000240248.55172.dd 16902165 16902165

[B36] Jiang D Liang J Noble PW Hyaluronan in tissue injury and repair Annu Rev Cell Dev Biol 2007 23 435 461 10.1146/annurev.cellbio.23.090506.123337 17506690 17506690

[B37] Stern R Asari AA Sugahara KN Hyaluronan fragments: an information-rich system Eur J Cell Biol 2006 85 699 715 10.1016/j.ejcb.2006.05.009 16822580 16822580

[B38] Serrano MC Pagani R Ameer GA Vallet-Regí M Portolés MT Endothelial cells derived from circulating progenitors as an effective source to functional endothelialization of NaOH-treated poly(epsilon-caprolactone) films J Biomed Mater Res A 2009 87 964 971 10.1002/jbm.a.31728 18257077

[B39] Bikfalvi A Cramer EM Tenza D Tobelem G Phenotypic modulations of human umbilical vein endothelial cells and human dermal fibroblasts using two angiogenic assays Biol Cell 1991 72 275 278 10.1016/0248-4900(91)90298-2 1724402 1724402

[B40] Koike N Fukumura D Gralla O Au P Schechner JS Jain RK Tissue engineering: creation of long-lasting blood vessels Nature 2004 428 138 139 10.1038/428138a 15014486 15014486

[B41] Auerbach R Lewis R Shinners B Kubai L Akhtar N Angiogenesis assays: a critical overview Clin Chem 2003 49 32 40 10.1373/49.1.32 12507958 12507958

